# Mental Wellbeing of Indonesian Students: Mean Comparison with UK Students and Relationships with Self-Compassion and Academic Engagement

**DOI:** 10.3390/healthcare10081439

**Published:** 2022-08-01

**Authors:** Yasuhiro Kotera, Jenai Lieu, Ann Kirkman, Kristian Barnes, Gillian H. T. Liu, Jessica Jackson, Juliet Wilkes, Riswani Riswani

**Affiliations:** 1 School of Health Sciences, University of Nottingham, Nottingham NG7 2HA, UK; yasuhiro.kotera@nottingham.ac.uk; 2 College of Health, Psychology and Social Care, University of Derby, Derby DE22 1GB, UK; jenai.lieu@gmail.com (J.L.); a.kirkman2@derby.ac.uk (A.K.); kmjbarnes@gmail.com (K.B.); gillianlht@gmail.com (G.H.T.L.); j.jackson2@derby.ac.uk (J.J.); 3 Faculty of Education and Teacher Training, State Islamic University of Sultan Syarif Kasim Riau, Pekanbaru 28293, Indonesia; riswani@uin-suska.ac.id

**Keywords:** Indonesian students, mental wellbeing, self-compassion, academic engagement, vigour, dedication

## Abstract

The number of Indonesian students in higher education has been increasing rapidly. However, many Indonesian university students report experiencing mental distress. Research on student wellbeing found that self-compassion (i.e., being kind towards oneself in challenging times) and academic engagement (i.e., a commitment and purposeful effort toward study) are essential to students’ mental wellbeing. With the present study, we aimed to assess the mental wellbeing of Indonesian students. A convenience sample of 156 Indonesian students completed self-report measures on mental wellbeing, self-compassion and academic engagement (consisting of vigour, dedication and absorption). To contextualise their mental wellbeing, data from Indonesian students were compared with those from 145 UK students using Welch *t*-tests. Correlation, regression and mediation analyses were then performed to examine the relationships among these variables. Indonesian students had higher levels of self-compassion and absorption and a lower level of dedication than UK students. Self-compassion and academic engagement explained 36% of variance in mental wellbeing. Self-compassion and vigour were identified as significant predictors of mental wellbeing, whereas self-compassion was the strongest predictor. Lastly, vigour did not mediate the pathway from self-compassion to mental wellbeing. Interventions to support self-compassion and academic engagement, especially vigour, in Indonesian students are recommended to support their mental wellbeing.

## 1. Introduction

### 1.1. Rapid Growth of Indonesian Higher Education

Indonesia is the fourth most populated country in the world, with a population of approximately 276 million people [1]. Indonesia is viewed as having a promising economic future and is predicted to become the world’s fourth largest economy by 2050 [2]. This rapid economic growth is positive; however, there are still socioeconomic problems within the country, with 27 million Indonesian people (more than 10% of the population) living on less than USD 0.75 a day [3]. Research has suggested that to allow Indonesia to experience full economic potential, the government needs to invest in key sectors, including education [4]. 

The Indonesian government has started to change their education policies to support its growing economy [5]. As a result, the number of Indonesian people attending university has grown rapidly. Decentralisation and increased government funding have supported a growth of 10 million Indonesian students since 2002 [6]. In 2016, 1.47 million students entered higher education (HE), almost doubling to 2.99 million by 2020 [7]. Additionally, the HE academic publication output has increased tenfold since 1996, with a 15% average annual growth (from 538 in 1996 to 5499 in 2014) [8]. The relationship between decentralisation and education development continues to be mutually reinforcing [9]. Investments in HE by the Indonesian government will ultimately contribute to increased work productivity, increased income and wealth and overall wellbeing in the general population [10]. 

### 1.2. Compromised Mental Wellbeing in Indonesian Students

Alongside a rapid increase in the number of students and scholarly output in Indonesian HE [11], the number of students claiming poor mental wellbeing has also increased. Consistent with findings in other countries [12,13,14], the mental wellbeing of Indonesian university students is compromised [15]. About a third to a half (37–53%) of undergraduate students in Indonesia reported high-stress symptoms [16], 25% experienced depression and 51% experienced anxiety [17]. In addition, high levels of loneliness have been reported [18]. Whereas the serious nature of depleted mental wellbeing among Indonesian university students has been clearly reported [15,17,19], information about the high financial cost of treatment, as well as the stigma surrounding mental health, support and treatment, is not readily available [20]. A lack of university mental health services and underutilisation of existing services is also common in non-Western countries [21], including Indonesia [22]. Overall, improved understanding of student mental wellbeing is needed in Indonesian HE. 

### 1.3. Self-Compassion

Self-compassion is associated with improved mental wellbeing [23]. It has been found to help students actualise adaptive achievement goals by allowing them to embrace their shortcomings and prioritise productive learning strategies [24]. Self-compassion is rooted in Buddhist teachings, which include a willingness to face and alleviate suffering [23]. Self-compassion has three elements that affect one’s wellbeing: treating oneself kindly during times of difficulty, recognising hardship and failure as a common human experience and using mindfulness to maintain a balanced awareness of painful thoughts and feelings [25,26]. Self-compassion acts as a mediator between positive psychological constructs and wellbeing [27] by enhancing resilience through the understanding and acceptance of one’s inadequacies [28]. A systematic review demonstrated that self-compassion is connected to a decrease in stress and depressive symptoms [29], as well as an increase in happiness and life satisfaction [30]. Self-compassion helps regulate emotions through the reduction of self-criticism, rumination and avoidance of painful experiences [25] and reduces the negative effects of stress on wellbeing [31,32,33]. Self-compassion is therefore important for mental wellbeing.

### 1.4. Academic Engagement

Academic engagement is associated with healthy mental wellbeing in higher education [34]. It refers to the commitment and purposeful effort by students in regard to their learning and other scholastic activities, which is measured by its components of vigour, dedication and absorption [35,36]. Vigour is defined as the willingness to persevere through difficulties represented as energy and mental agility; dedication is defined as investment in one’s work, resulting in inspiration and enthusiasm; and absorption is defined as the sense of immersion in one’s work [37]. These components of engagement can positively impact students’ cognitive appraisals of their academic experience through resource building and implementation [36], thus influencing their probability of increased motivation and academic performance [38]. Academic engagement in postsecondary education has been considered a determining factor for students’ achievement and personal development [39]. A longitudinal survey of undergraduate students based in the UK showed that engagement and wellbeing were positively related, also suggesting a feedback loop of increased engagement, increased academic performance and increased wellbeing, leading again to increased engagement [40]. Overall, engagement leads to increased satisfaction in postsecondary learning, leading to improved wellbeing.

### 1.5. The Effect of Academic Engagement on Self-Compassion and Mental Wellbeing

Academic engagement and self-compassion are both positively associated with mental wellbeing [32,40] and share similarities despite little empirical evidence comparing the two. Research revealed that students who are passionate and engaged in their academic studies and who exhibit resilience toward challenges and compassion toward themselves have a high level of wellbeing [41]. Self-compassion is associated with student engagement and the ability to adapt achievement goals, which allows students to confront failure and adopt healthy and productive learning ethics [24]. Academic engagement and self-compassion both facilitate mental wellbeing through association with increased resilience and personal development [24,39,42]. Students who practice self-compassion and who are academically engaged are more resourceful when managing their goals and motivation [36], which promotes a positive effect on their wellbeing. This suggests that engagement and self-compassion share similarities in terms of facilitating positive mental wellbeing by enhancing students’ ability to navigate difficult circumstance through positive cognitive appraisal [36]. Therefore, in the present study, we evaluate whether academic engagement could mediate the pathway from self-compassion to positive mental wellbeing.

### 1.6. Comparison with UK Students

In this study, the mean scores of mental wellbeing among Indonesian students were compared with those of UK students in order to (a) contextualise mental wellbeing in Indonesian students and (b) discuss how cultures may influence mental wellbeing. When comparing cross-cultural differences between postsecondary students in the UK and Eastern countries, such as Indonesia, the UK scored significantly high in individualism [43], relatively high in masculinity [43] and low for power distance in the school system [44]. Regarding Hofstede’s cultural dimensions theory [43], the cultural dimension of individualism–collectivism involves societal orientations, masculinity–femininity identifies societal views and power distance explores unequal distributions of power between individuals. Hereafter, we will use the words ‘success-driven’ and ‘quality-oriented’ instead of ‘masculine’ and ‘feminine’ for accuracy and clarity [45]. UK culture is highly individualistic, and it emphasises personal achievement and independence [43], compared to collectivist cultures that emphasise group achievement and interdependence [46]. UK culture scored relatively high for the dimension of success-drive, indicating the society’s tendency to prioritise accomplishment and achievement over caring for others and maintaining a high quality of life [43]. Lastly, UK culture scored low in power distance, suggesting that there is a value in equal treatment among individuals [43]. Low power distance in the school system is demonstrated through learner-centred environments with increased interaction between students and teachers, compared to the high levels of power distance common in Eastern culture school systems [44]. By comparing the mental wellbeing levels between these culturally contrasting groups, we aimed to inform how cultures may explain the level of difference in mental wellbeing. Culturally aware approaches to wellbeing are needed in modern higher education [47]. 

### 1.7. Study Aims

The aim of this study was to evaluate the mental wellbeing of Indonesian students, along with self-compassion and academic engagement, namely vigour, dedication and absorption. First, the levels of these five constructs (mental wellbeing, self-compassion, vigour, dedication and absorption) were assessed by comparing them with UK university students (Aim 1). Second, the extent of variance in mental wellbeing could be predicted by self-compassion, vigour, dedication and absorption, and significant predictors of mental wellbeing were identified (Aim 2). Lastly, we examined how engagement mediates the pathway from self-compassion to mental wellbeing (Aim 3). 

## 2. Materials and Methods

### 2.1. Participants

Students >18 years studying a caring profession subject (counselling and education) full-time at a university in Indonesia were eligible for this study. Caring profession subjects relate to occupations in which humans take care of other humans [48], including the allied health professions, counselling and education [49]. All study materials were provided as hard copies, prepared in English and disseminated by an independent tutor to 162 students (October–December 2021), meaning the study had a 96% (*n* = 156) response rate. The majority of respondents were female (*n* = 128), with 25 males and 3 students who did not disclose their gender. Participants ranged in age from 18 to 22 years (19.07 ± 0.98). Five students were postgraduate students. Our sample was similar in age to the general Indonesian student population, but was more female-dominated (age: 20 years old, 49% female [50]). The study received ethical approval from the co-author, R.R.’s, university (State Islamic University of Sultan Syarif Kasim Riau: Ref KE/KEP-FPP/01/05/2022) and involved no deception or financial incentives. 

Students were provided with supporting information about mental health during the study. Data were compared with data obtained in a study involving students studying a caring profession subject in a UK university (*n* = 145; entire sample), using the same participation criteria; the original studies were published previously [41,51] (University of Derby: Ref 011017YK). As with the Indonesian sample, a paper survey was disseminated by an independent tutor. No participation incentive was offered. Data were collected between April and May 2018. In this UK study, the majority of students were also female (*n* = 130), with 15 male students. They had a wider age range of 17–52 years (26.80 ± 8.64), with 133 undergraduates and 12 postgraduates. The respondent age range in the UK study reflects the wider trend that caring professions attract both young and mature students [52]. The younger age range of the Indonesian respondents reflects the wider student population of the country. The predominance of female participants in both study samples is consistent with the global trend within health and social care, which consists of a largely female workforce [53]. Table 1 summarises the demographic information of both samples.

### 2.2. Materials

The materials consisted of three validated scales to measure students’ mental wellbeing, self-compassion and engagement in academia. The original English version of all three scales had not been previously used in Indonesian samples. Mental welling was measured using the established seven-item Short Warwick–Edinburgh Mental Wellbeing Scale (SWEMWBS; [54]). This scale was selected for its holistic evaluation of mental wellbeing and high internal consistency [55]. Respondents were asked to make both hedonic and eudemonic reflections on the past two weeks on a five-point Likert scale (e.g., ‘I’ve been dealing with problems well’; 1 = ‘None of the time’ to 5 = ‘All of the time’). The internal consistency of the SWEMWBS is high (α = 0.85; [56]). 

Self-compassion was measured using the 12-item Self-Compassion Scale–Short Form because of its high internal consistency (SCS-SF; [57]). Scale items are measured on a five-point Likert scale and include questions such as, ‘I try to be understanding and patient towards those aspects of my personality I don’t like’ and measures whether, in difficult situations, a respondent is consistently kind to themselves (0 = ‘Almost never’ to 5 = ‘Almost always’). SCS-SF demonstrates high internal consistency (α = 0.86 [57]). 

Engagement was measured to consider how confident and active students are in their academic workload, using the 17-item Utrecht Work Engagement Scale (UWES-S) because the scale (global score) and subscales demonstrate a good-to-high internal consistency (α = 0.63–0.81; [58]). The scale consists of three subscales—vigour, dedication and absorption—and each item is measured on a seven-point Likert scale. Vigour relates to the student’s mental capacity, which leads to substantial effort in academia, for example, ‘I am very resilient, mentally, as far as my studies are concerned’. Dedication relates to the student’s commitment to academia, for example, ‘My study inspires me’. Absorption relates to the student’s immersion in academia, for example, ‘When I am studying, I forget everything else around me’ [37]. 

### 2.3. Analysis

Data were screened and tested for assumptions and outliers with parametric tests, correlations and a multiple regression model using SPSS (v25) and Process Macro version 3 [59]. An additional path analysis was performed in order to identify whether an engagement component could mediate the pathway from self-compassion to mental wellbeing. 

### 2.4. Results

#### 2.4.1. Levels of Mental Wellbeing, Self-Compassion and Academic Engagement (Aim 1)

An independent-samples t-test was conducted to compare the levels of mental wellbeing, self-compassion and academic engagement—namely, vigour, dedication and absorption—between Indonesian and UK students (Table 2). According to Levene’s test for equality of variances, the assumption of homogeneity of variances was not maintained for self-compassion (*p* < 0.001) and absorption (*p* < 0.001); thus, Welch t-tests were used.

Indonesian students had higher levels of self-compassion (mean difference, −0.45; CI 95% [−0.59, −0.32], *t*(261.07) = −6.55, *p* < 0.001, *d* = −0.77) and absorption (mean difference, −0.66, CI 95% [−0.92, −0.39], *t*(293.19) = −4.81, *p* < 0.001, *d* = 0.93) and a lower level of dedication (mean difference, 0.88, CI 95% [0.67, 1.10], *t*(293.19) = 8.06, *p* < 0.001, *d* = −0.56) than UK students. There were no significant differences in mental wellbeing (*p* = 0.17) and vigour (*p* = 0.28). 

#### 2.4.2. Prediction of Mental Wellbeing (Aim 2)

Multiple regression analyses were conducted to identify predictors of mental wellbeing. After adjusting for age and gender (Step 1), self-compassion, vigour, dedication and absorption were entered as predictor variables (Step 2), and mental wellbeing was entered as an outcome variable (Table 3). Multicollinearity was of no concern (variance inflation factors < 10). 

These four predictor variables accounted for 36% of mental wellbeing, indicating a large effect size [60]. Self-compassion and vigour were significant positive predictors of mental wellbeing, whereas self-compassion was the strongest predictor of mental wellbeing.

#### 2.4.3. Mediation of the Self-Compassion–Mental Wellbeing Pathway (Aim 3)

**Figure 1:**
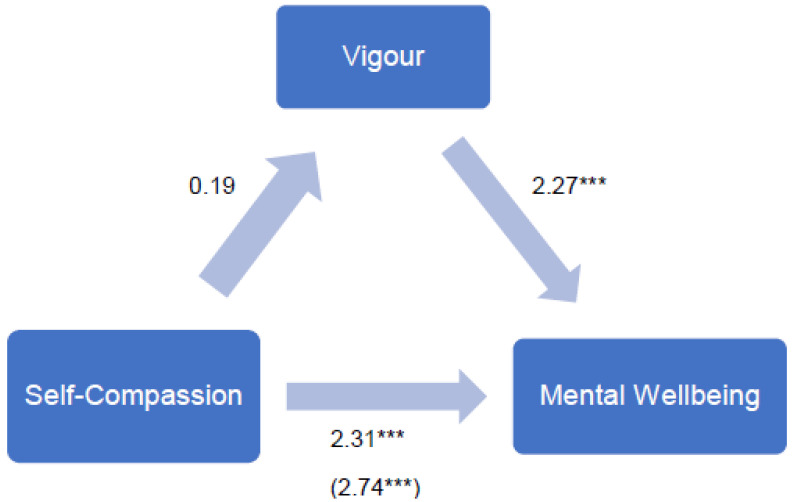
Evaluating whether vigour mediates the pathway from self-compassion to mental wellbeing. Parallel mediation model: self-compassion as a predictor of mental wellbeing, mediated by vigour. The confidence interval for the indirect effect is a BCa bootstrapped CI based on 1000 samples. *** *p* < 0.001; direct effect (total effect).

Lastly, to evaluate whether academic engagement mediates the pathway from self-compassion to mental wellbeing, path analyses were conducted using model 4 in the Process macro (parallel mediation model; [59]) (see Figure 1). Among the three subscales of academic engagement, vigour was used, as it was found to be a significant predictor of mental wellbeing.

Vigour did not mediate the pathway from self-compassion to mental wellbeing, as the path from self-compassion to vigour was not significant (*b* = 0.19, *t*(154) = 1.23, *p* = 0.22). However, the total effect of self-compassion on mental wellbeing, including vigour, was significant (*b* = 2.74, *t*(154) = 4.13, *p* < 0.001). The direct effect of self-compassion on mental wellbeing, controlling for vigour, was also significant (*b* = 2.31, *t*(153) = 4.05, *p* < 0.001). 

## 3. Discussion

This study assessed the mental wellbeing of Indonesian students. Indonesian students had higher levels of self-compassion and absorption and a lower level of dedication than UK students. Self-compassion and academic engagement explained 36% of variance in mental wellbeing, and self-compassion and vigour were identified as predictors of mental wellbeing, where self-compassion was the strongest predictor. Lastly, vigour did not mediate the pathway from self-compassion to mental wellbeing. 

The significant difference in the levels of self-compassion between Indonesian and UK students observed in this study may be explained by cultural differences [61,62]. The higher self-compassion in Indonesian students can be attributed to their lower scores in Hofstede’s cultural dimensions of individualism and success-drive [63]. Montero-Marin et al. [62] reported that individualism and success-drive have a moderate effect on negative items of self-compassion. More individualistic and success-driven cultural values create a more competitive system, evoking social comparison [64]. Because people from these cultural backgrounds are more self-referent to success, they try to avoid failures and are therefore more self-critical [62]. Indeed, the UK is highly individualistic and success-driven, and society emphasises personal success and achievement [43]. This characteristic of the UK’s cultural value is aligned with the result of having a lower self-compassion score than that of Indonesian students. Moreover, it is noteworthy that despite displaying higher levels of self-compassion than UK students, the mental wellbeing of Indonesian students was similar to that of UK students. Previous research suggested that self-compassion mediates pathways from stress to psychopathologies [65]. It is possible that high stress in Indonesian society relating to the rapid growth in domains such as education and economy impacted students’ mental wellbeing, despite the mediative effects of self-compassion. Future research is needed to understand the first-hand experience of Indonesian students. Interview-based research focusing on mental wellbeing constructs [66] is suitable to deeply appraise internal experiences.

Absorption scores were significantly higher in Indonesian students than UK students. This may also be explained by cultural differences between the two countries. The power distance in Indonesia is higher than that in the UK [63], with people in Indonesia, in general, being more accepting of social hierarchy. Their high-power distance may help Indonesian students to focus less on competing with each other and more on enjoying their studies. A high level of absorption in studies has been reported in Indonesian students [67,68]. 

Conversely, UK students’ low power distance and high success-drive can explain their higher level of dedication relative to Indonesian students. Contrary to the inner quality of absorption, dedication relates more to the external; it is about the investment put into the work [37], relating to a sense of significance, challenge and pride taken in study [69]. Success-driven societies value personal accomplishment; therefore, students tend to commit more to their studies in order to achieve future success [43]. Low power distance can be another drive to enhance dedication. As students are able to interact with teachers more easily, they may find academic activities more meaningful, resulting in increased motivation to attain high achievement [44,70]. However, in higher power distance societies, instructions are usually given from an authority, causing students to follow passively [71]. The strong hierarchy may limit students’ participation and motivation to commit, hence low dedication. 

Of the four predictor variables, self-compassion and vigour were significant positive predictors of mental wellbeing, with self-compassion as the strongest predictor. This finding supports previous research in student populations [23,72] and suggests that self-compassion training is critical to students maintaining positive mental wellbeing. Self-compassion training helps students improve overall wellbeing by reducing academic stress and anxiety [10,73,74]. It does so by equipping students with techniques to help them acknowledge and accept their weaknesses and to develop more effective learning strategies [24]. Several studies support the positive impact of self-compassion training on mental wellbeing [75,76]. Self-compassion interventions have also been shown to have a sustained longer-term positive mental wellbeing effect [30,75,76]. In particular, the practice of mindfulness has been found to be an effective format for developing self-compassion [76,77]. Beaumont et al. [78] recommend that schools and universities develop a culture of compassion to protect students’ mental wellbeing. They suggest that self-compassion training should not be an add on but an integral part of the taught curriculum. Implementation and evaluation of self-compassion training for Indonesian students are needed.

Lastly, vigour, although the second strongest predictor of wellbeing, did not mediate the pathway from self-compassion to wellbeing, suggesting that vigour’s contribution to wellbeing occurs through a different pathway. One possible explanation for this finding is the mediation model we used, which assessed vigour separately from the other academic engagement components. Vigour is argued to predict high academic achievement [79] and is associated with efficacy [80]. However, vigour without dedication can lead to academic burnout [81] and a lack of motivation [82]. This suggests that dedication could be a pathway mediator from vigour to wellbeing, although further research is required to affirm this. 

Vigour interventions focused on improving personal resources (e.g., self-efficacy) and physical movement have been shown to increase vigour [83] and are suggestive of effective types of vigour training. Vigour training has been suggested to help PhD students mediate burnout [84]. Importantly, research has indicated that first-year degree students have higher levels of vigour and dedication, which decrease in subsequent years [85]. This offers insight into the potential timing of vigour training in a student’s life cycle for maximum effectiveness. 

## 4. Limitations

Firstly, in the present study, we employed convenience sampling at one university, limiting the generalisability. Secondly, the comparisons were made between counselling and education students in Indonesia and counselling and occupational therapy students in the UK. Comparing students solely from a single discipline could contribute to a more accurate discussion of cultural differences (however, it can be difficult or impossible to do so in cross-national research). Additionally, in this study, we did not consider other aspects that could have enabled more accurate comparisons to be made, such as ethnicity, religion and socioeconomic status. Thirdly, the cross-cultural use of self-report measures might have limited the accuracy of our results [86]. Moreover, the accuracy of the SCS-SF scale used in this study has been questioned and is currently under debate [87]. However, as the scale has been used broadly among university student samples [88], we felt it was appropriate for use in the present study. Fourthly, the causal directions of the variables were not assessed. Lastly, although the study was conducted when COVID-19 cases were declining steadily in Indonesia [89], the impact of the COVID-19 pandemic was not discussed. Furthermore, the UK data were collected before the outbreak of the COVID-19 pandemic. Indonesian universities tried to mitigate the spread of the COVID-19 infection, including by limiting face-to-face teaching [90]. Resulting social isolation compromised the mental wellbeing of Indonesian students [91]. The impact of COVID-19 on the study variables needs to be evaluated. 

## 5. Conclusions

As Indonesian higher education continues to develop rapidly, many Indonesian university students suffer from compromised mental wellbeing. In the present study, we identified that Indonesian students had higher levels of self-compassion than UK students. Self-compassion and vigour were identified as significant predictors of mental wellbeing. Our findings will help researchers and educators in Indonesia to effectively support the mental wellbeing of students in higher education. 

## Figures and Tables

**Table 1:** Demographic data for 156 Indonesian students and 144 UK students.

	Indonesian Students		UK Students	
Age	Years		Years	
Mean	19.07		26.8	
SD	0.98		8.64	
Range	18–22		17–52	
Gender	*n*	*%*	*n*	*%*
Female	128	82.05%	130	89.66%
Male	25	16.03%	15	10.34%
Unknown	3	1.92%	0	0.00%
Level of Study	*N*	*%*	*N*	*%*
Undergraduate	151	96.79%	133	91.72%
Postgraduate	5	3.21%	12	8.28%

**Table 2:** Comparing the levels of mental wellbeing, self-compassion and academic engagement (vigour, dedication and absorption) between 156 Indonesian students and 144 UK students.

	Indonesian Students			UK Students							
Variable	M	SD	*α*	M	SD	*α*	*t*	MD	CI 95% [L, U]		*d*
Mental Wellbeing	23.43	4.30	0.72	23.94	4.86	0.85	0.96	0.51	−0.54	1.55	0.11
Self-Compassion ***	3.32	0.50	0.67	2.87	0.67	0.84	−6.55	−0.45	−0.59	−0.32	−0.77
Vigour	3.63	0.94	0.72	3.70	1.08	0.76	0.60	0.07	−0.16	0.30	0.07
Dedication ***	3.87	0.93	0.60	4.75	0.97	0.63	8.06	0.88	0.67	1.10	0.93
Absorption ***	3.78	0.97	0.79	3.13	1.35	0.80	−4.81	−0.66	−0.92	−0.39	−0.56

*** *p* < 0.001 *α* = Cronbach’s alpha significant difference between the two groups according to Welch *t*-tests.

**Table 3:** Multiple regression: self-compassion, vigour, dedication and absorption for mental wellbeing in 156 Indonesian students.

Outcome: Mental Wellbeing					
	B	SE_B_	b	95% CI [L, U]	
Step 1					
Gender	−1.12	0.86	−0.11	−2.82	0.58
Age	−0.34	0.36	−0.08	−1.05	0.37
Adj. R^2^	0.2%				
Step 2					
Gender	−1.30	0.69	−0.12	−2.67	0.06
Age	−0.38	0.29	−0.09	−0.96	0.20
Self-Compassion ***	2.14	0.59	0.25	0.98	3.30
Vigour *	1.23	0.54	0.27	0.17	2.29
Dedication	0.76	0.49	0.16	−0.22	1.73
Absorption	0.63	0.62	0.14	−0.60	1.86
Δ Adj. R^2^	36%				

*** *p* < 0.001, * *p* < 0.05.

## Data Availability

The data presented in this study are available on reasonable request from the corresponding author. The data are not publicly available due to ethical restrictions.
